# Prospective study of dietary mushroom intake and risk of mortality: results from continuous National Health and Nutrition Examination Survey (NHANES) 2003-2014 and a meta-analysis

**DOI:** 10.1186/s12937-021-00738-w

**Published:** 2021-09-21

**Authors:** Djibril M. Ba, Xiang Gao, Laila Al-Shaar, Joshua Muscat, Vernon M. Chinchilli, Paddy Ssentongo, Xinyuan Zhang, Guodong Liu, Robert B. Beelman, John P. Richie

**Affiliations:** 1 Department of Public Health Sciences, Pennsylvania State University College of Medicine, Hershey, PA USA; 2 Department of Nutritional Sciences, Pennsylvania State University, University Park, PA USA; 3 Department of Food Science and Center for Plant and Mushroom Foods for Health, College of Agricultural Sciences, Pennsylvania State University, University Park, PA USA

**Keywords:** Mushroom, Mortality risk, Diet, Prospective study, Meta-analysis, NHANES

## Abstract

**Background:**

Whether mushroom consumption, which is a rich source of potent antioxidants ergothioneine and glutathione, vitamins, and minerals (e.g., selenium & copper), is associated with a lower mortality risk is not well understood. This study aimed to examine the association between mushroom consumption and risk of mortality in a prospective cohort study and a meta-analysis of prospective cohort studies.

**Methods:**

We followed 30,378 participants from the continuous National Health and Nutrition Examination Survey (NHANES) extant data (2003-2014). Dietary mushroom intake was assessed using up to two 24-h recalls. Mortality was evaluated in all participants linked to the National Death Index mortality data through December 31, 2015. We used Cox proportional hazards regression models to calculate multivariable-adjusted hazard ratios (HRs) and 95% confidence intervals (95% CIs). We also conducted a meta-analysis, including results from our present study and 4 other cohort studies.

**Results:**

During a mean (SD) of 6.7 (3.4) years of follow-up, a total of 2855 death cases were documented among NHANES participants. In our analysis of continuous NHANES, we found a non-significant association between mushroom consumption and all-cause mortality (adjusted hazard ratio (HR) = 0.84; 95% CI: 0.67-1.06) after adjusting for demographic, major lifestyle factors, overall diet quality, and other dietary factors, including total energy. The meta-analysis of prospective cohort studies, including 601,893 individuals, showed that mushroom consumption was associated with a lower risk of all-cause mortality (pooled risk ratio: 0.94; 95% CI: 0.91, 0.98).

**Conclusion:**

In a meta-analysis of prospective cohort studies, mushroom consumption was associated with a lower risk of all-cause mortality.

**Supplementary Information:**

The online version contains supplementary material available at 10.1186/s12937-021-00738-w.

## Background

Unhealthy dietary intake such as low intake of fruits, vegetables, high sodium intake, saturated fats, and added sugars has been classified as the leading factor contributing to death, risk of major chronic diseases, cancer, and health complications [1, 2]. As a consequence, inadequate dietary intake constitutes a significant public health threat worldwide, including in the United States (US). A previous study suggested that improvement of dietary intake such as healthy eating could prevent 1 in every 5 death globally [3].

Although mushrooms are categorized as other vegetables in the US Department of Agriculture (USDA) food groups and share some nutritional characteristics with plant-derived foods, they are neither a plant nor animal but scientifically belong to the fungal kingdom [4, 5]. Mushrooms have been a part of the human diet for centuries because of their unique taste and role in a healthful diet for being low in energy, sodium, and fats; they are also cholesterol- and gluten-free [6–9]. They are a good source of many bioactive compounds, including phytochemicals [10, 11], polysaccharides (β-glucan) [12], minerals (selenium and copper) [13, 14], essential vitamins (e.g., niacin, thiamin, riboflavin, and vitamin C) [15–17], which fit well in the healthy eating pattern and healthy aging strategy [18]. They are also a good source of ergocalciferol (vitamin D_2_) when exposed to UV light during the growing process [15].

Mushrooms are also rich sources of powerful antioxidants ergothioneine and glutathione, which play a significant role in preventing chronic diseases and mortality [19–23]. Ergothioneine is an amino acid with a unique chemical structure produced by certain fungi and a few mycobacteria but not by animals or higher plants [24–26]. Consequently, ergothioneine is obtained exclusively through dietary sources, with mushrooms having the highest levels compared to other foods [4, 7, 20, 23, 27, 28].

Mushrooms have often been informally categorized into broad categories in diet assessment. They have been considered a “forgotten source of nutrients” [29], making it hard to calculate their actual consumption and contribution to human health. Despite this limitation, accumulating evidence suggests that mushroom consumption may be associated with a lower risk of chronic diseases, including cancers [30], metabolic syndrome, [22] cognitive impairment, [31, 32], and dementia [33]. A few epidemiological observational studies also have reported an inverse association between mushroom consumption and the risk of mortality [34, 35]. However, other epidemiological studies that have examined the effects of mushroom intake on mortality risk have yielded non-significant associations [36, 37]. Given the inconsistent findings in the literature and a lack of a comprehensive meta-analysis, we, therefore, examined the association of mushroom consumption with all-cause and cause-specific mortality using the continuous National Health and Nutrition Examination Survey (NHANES) 2003-2014. We also conducted a meta-analysis that includes relevant published data on mushroom consumption and all-cause mortality combined with results from the present continuous NHANES. We hypothesized that mushroom consumption is associated with a lower risk of all-cause mortality.

## Methods

### Analysis of continuous NHANES data

#### Data source and study design

We invoked a prospective cohort study using publicly available de-identified continuous NHANES 2003-2014 and meta-analysis of 5 prospective cohort studies. Total and cause-specific mortality were assessed in all participants linked to the National Death Index (NDI) mortality data from 2003 through December 31, 2015. The NHANES surveys were conducted by the National Center for Health Statistics (NCHS) of the Centers for Disease Control and Prevention (CDC). NHANES is a complex, multistage, probability sampling design that allows results to be extrapolated to the entire 50 states of the US, including the District of Columbia. The program is designed to assess the health and nutritional status of the US civilian, non-institutionalized population [38]. Detailed information regarding the NHANES Laboratory/Medical Technologists Procedures and Anthropometry Procedures has been described previously [39]. The NCHS Research Ethics Review Board approves the survey protocol, and all participants or their proxies provided signed informed consent [38]. Detailed information about the dietary recall interview portion of the survey has been published previously [40].

Given that all NHANES data are de-identified and available in the public domain, the Institutional Review Board at the researchers’ institution does not consider this to be human subject research. Therefore, human subjects’ approval was not necessary for the present study.

### Study population

The present study included individuals aged 18 years or older from a nationally representative sample of continuous NHANES 2003-2014 with data on mortality status (*n* = 35,848). Participants without reliable dietary intake data (*n* = 4226) were excluded from the present study. Furthermore, as done by a previous study [41], we also excluded individuals who reported implausible daily energy intake levels (< 800 kcal or > 4200 kcal for men and < 500 kcal or > 3500 kcal for women) (*n* = 1244), leaving a total of 30,378 participants for the final analysis of continuous NHANES.

### Assessment of mushroom consumption

Beginning 2003, NHANES participants were eligible for up to two 24-h dietary recall interviews in which respondents reported all foods and beverages consumed during the preceding 24-h. The Day 1 dietary recall interview was collected in person in the Mobile Examination Center (MEC) by trained interviewers. The Day 2 dietary recall was collected by telephone 3 to 10 days after the MEC interviews. Both 24-h dietary recalls were collected using the computerized US Department of Agriculture (USDA) Automated Multiple-Pass Method [38]. Detailed information about the types and amounts of individual foods reported by each participant, including foods containing mushrooms (reported as grams/day), were obtained from the NHANES Individual Foods Files (IFF) using the USDA food codes, 8-digit numbers that identify foods in the Nutrient Databases for Dietary Studies (FNDDS). Detailed information about NHANES dietary data and the IFF can be found on the NHANES website (https://wwwn.cdc.gov/nchs/nhanes/search/datapage.aspx?Component=Dietary). As done by previous studies [18, 34], mushroom intake was defined as the consumption of any amount of mushrooms using the USDA food codes including foods that were mixed dishes with mushrooms, for example, egg omelet or scramble egg served with mushrooms, or mushrooms alone, for example, raw mushrooms. Since mushrooms are frequently incorporated into mixed dishes (supplementary Table 1), the current analysis separated out mushrooms in mixed dishes. The US Environmental Protection Agency-USDA Food Commodity Intake Database (FCID) commodity codes, which reports intake amounts per 100 g of food, was used to determine the actual amounts of mushroom intake as follows: grams of intake by food code multiplied by the commodity weight of the mushroom contribution from FCID per 100 g of the food code [18]. Detailed information regarding the Food Commodity Intake Database is described elsewhere [42]. Unique USDA food codes used to identify mushroom consumers for this study (*n* = 1345) are presented in the supplemental Table 1. Only individuals with reliable and complete dietary records for mushroom intake status as determined by NCHS were included in the current analysis.

### Mortality ascertainment

The primary outcome of interest for this study was all-cause mortality, ascertained by the NCHS using death certificates. We also assessed cause-specific mortality as an exploratory analysis. The de-identified data of continuous NHANES 2003-2014 participants were linked to the Mortality Files linked through December 31, 2015, with a probabilistic matching algorithm to the NDI to ascertain mortality status using the NHANES unique sequence number [43]. Participants with no match to the mortality file were assumed to be alive during the follow-up period. All-cause mortality in the current analysis included all specified causes of death recorded in the Public-use Linked Mortality files. The underlying cause of death was coded using the international classification of diseases, 10th revision (ICD-10). Detailed information about the linkage methods has been reported previously [43].

### Assessment of covariates

Based on a previous study [34], the following covariates were included in our analysis to reduce potential confounding age (years), sex (men/women), ethnicity-race (Mexican American, other Hispanic, Non-Hispanic White, Non-Hispanic Black, other race), education (non-college degree/college degree or above), marital status (never married/married), BMI (< 24.9, 25.0-29.9, ≥30), smoking status (never smoker, ever smoker), physical activity (somewhat active, active and very active), alcohol (g/d) intake, energy-adjusted carbohydrates (g)/1000 kcal/d), fiber (g)/1000 kcal/d), total energy intake (kcal/d), and the Healthy Eating Index-2010 (HEI-2010) score, a measure of diet quality with a higher score indicating better diet quality. The HEI-2010 score included 12 components scales (range 0-5, 0-10, or 0-20), which are combined to create a total HE-2010 score (range from 0 to 100) [44]. The 12 components of the HEI-2010 include total fruit, whole fruit, total vegetables, greens & beans, whole grains, dairy, total protein foods, seafood and plant proteins, FAs, refined grains, sodium, and empty calories [45]. The HEI-2010 total score was calculated using the Food Pattern Equivalents Database (FPED) and MyPyramid Equivalents Database 2.0 (MPED 2.0) and publicly available SAS macro code from the National Cancer Institute website (https://epi.grants.cancer.gov/hei/sas-code.html). Carbohydrates and fiber were included as covariates because they are related to all-cause mortality [46, 47]. Measures of physical activity were calculated based on the 3 domains in which physical activity is performed, such as leisure-time physical activity (i.e., sports and recreational activities), transportation-related physical activity (i.e., bicycling and walking), and domestic physical activity (i.e., work-related physical activities). A total physical activity score metabolic equivalents of task (MET)-minutes/week was calculated by summing the total MET-minutes from each domain. The total score MET-minutes/week was categorized into three groups: < 500 MET-minutes/week (somewhat active), 500-999 MET-minutes/week (active), ≥1000MET min/week (very active). These cut-points are based on their equivalence to the physical activity guideline— < 500 MET min/week reflects activity equivalent below the minimal guideline, 500 MET min/week is equivalent to the minimal guideline, and ≥ 1000 MET min/week is equivalent to double the minimal guideline [48, 49].

### Power analysis and sample size

We conducted a power calculation, and the results show that with a ratio between the unexposed (non-mushroom consumers) and exposed (mushroom consumers) of 21.6 and mortality risk among non-mushroom consumers of 0.09% and a maximum number of 1345 mushroom consumers, the statistical power for the study would be 98, 82.6, and 57% for a HR of 0.70, 0.78, and 0.84, respectively, assuming a Type I error of 5%.

### Statistical analysis

Analyses were conducted using appropriate sample weights, clustering, and stratification as specified by the NCHS for analysis of NHANES data to account for the complex sampling design [38]. For each participant, the person-time was calculated as the time from the baseline survey participation interview date until the date of death or end of follow-up (December 31, 2015), whichever came first. Univariable analyses were conducted using the Rao-Scott χ2 test for categorical variables and t-test for continuous variables. We used time-dependent multivariable Cox proportional hazards models (proc surveyphreg; SAS institute) to calculate hazard ratios (HR) with 95% confidence intervals (CI) for the association between mushroom consumption and risk of mortality, and the proportional hazards assumption was not violated. Models were adjusted for the covariates mentioned above. To assess whether there is evidence of a linear dose-response relationship between greater mushroom consumption and all-cause mortality, we further categorized mushroom intake into 4 groups: no mushroom intake (0 g/d, *n* = 29,033), lowest (median intake = 5.1 g/d, range = 10.9, *n* = 717), middle (median intake = 18.1 g/d, range = 17.3, *n* = 398), and highest (median intake = 39.2 g/d, range = 146.7, *n* = 230). Tests for linear trend were examined for significance by using the median value for each group of mushroom intake, which was then analyzed as a continuous variable in the multivariable-adjusted Cox model [50]. The interaction between mushroom intake and age, ethnicity-race, sex in association with total mortality were statistically tested by including the interaction terms in the Cox proportional hazards regression models. Imputation was performed for participants with missing demographic and lifestyle variables using the fully conditional specification method [51]. Variance Inflation Factor (VIF) was used to assess multicollinearity, leaving only variables in the final model (3) with a VIF value of 3 and less.

To further test the robustness of our results, we conducted a series of sensitivity analyses. First, to minimize potential bias, we additionally adjusted for a propensity score. Since the propensity model aims not to make inferences to the US non-institutionalize population, thus the propensity score model was estimated using unweighted logistic regression by including the covariates mentioned above in the final model plus the survey weights [52]. The propensity score approach allows us to balance baseline data between participants with mushroom intake and those without mushroom intake. Therefore, including the survey weight in the model as a covariate may thus improve the assumption of unconfounded treatment assignment [53]. Second, to understand the short- vs. long-term impact of mushroom intake on mortality, a 2-year lag analysis was conducted to calculate hazard by excluding mortality cases occurring during the first 2 years of follow-up. Third, because major chronic diseases are strongly associated with the risk of mortality [54], we conducted a sensitivity analysis by excluding participants with baseline CVD, diabetes, and cancer. Statistical analyses were performed using SAS statistical software version 9.4 (SAS Institute, Cary, NC, USA). Statistical tests were reported as significant at *p* values less than 0.05.

### Meta-analysis

We conducted a meta-analysis that included findings from previous prospective cohort studies that reported risk estimates for all-cause mortality by mushroom consumption. We performed a systematic literature search in PubMed (MEDLINE), Web of Science, and Cochrane Library databases to identify relevant prospective cohort studies on the association between mushroom consumption and the risk of all-cause mortality published from January 1, 1966, up to May 1, 2021. The following keywords were used: “Mushroom” OR “Mushrooms” OR “Agaricales” AND “Prevention” OR “risk OR risks” AND “Mortality” OR “Death.” In addition, we manually searched the references list of the selected articles and relevant reviews. Only articles written in English-language were included in the present meta-analysis. The search process is delineated in Supplemental Figure 1. The included studies met the following criteria: (1) used an observational study design (cohort study design); (2) dietary mushroom intake as exposure; (3) total mortality as the outcome of interest; (4) relative risks (RRs) or hazard ratios (HRs) with 95% confidence interval (CIs). One study reported separate RRs for men and women [37]. In this situation, we used the random-effects models to pool the RRs within that specific study.

The data extraction was done by 2 authors independently. Disagreements were resolved by discussion with an available third co-author in order to reach a consensus. The following data were extracted from each publication: the first author’s name, the year of publication, sex, sample size, dietary assessment, outcome assessment, the country in which the study was conducted, study design, mortality status, the mean age of study participants, number of cases, categories of mushroom consumption, reported HRs or RRs with corresponding 95% CIs, duration of follow-up, and the covariates adjusted for in the final multivariable regression models We first log-transformed all the reported effect sizes of data to normalize the distributions. To examine the associations between mushroom intake and the risk of total mortality, we pooled the RR data from each study, weighted by the inverse of their variances. The *metagen* function from the R package meta was used to calculate the pooled effect estimates using random-effects models, which account for between and within study variabilities [55]. Random-effects models were pooled using DerSimonian and Laird’s method for the association between mushroom intake and the risk of total mortality. Individual and pooled estimates were graphically presented in forest plots. Potential heterogeneity between studies was quantified using Cochran’s *Q* test and *I*^*2*^ statistics expressed as a proportion (%) [56]. A *p*-value of *P* < 0.05 was used to determine the level of significance of heterogeneity. Since the total number of our studies included in the meta-analysis was less than 10 studies, it is not very reliable to assess publication bias using the Begg or Egger tests and visual inspection of a funnel plot [57]. We reported the meta-analysis per  the Preferred Reporting Items for Systematic reviews and Meta-Analyses (PRISMA) guidelines and the guidelines established for reporting nonrandomized studies in Cochrane Library [58, 59]. Meta-analysis was conducted using R version 3.6.2 (R Foundation for Statistical Computing, Vienna, Austria).

## Results

### Analysis of continuous NHANES results

A total of 30,378 participants (the mean age 45.9 ± 0.3 y) were included in the current analysis. More than half of the participants were women 15,884 (52.5%); 13,966 participants (68.6%) were non-Hispanic White, and 14,843 (58.3%) had a college degree or higher (Table 1). Compared with individuals without mushroom intake, a higher proportion of mushroom consumers were women, non-Hispanic White, and had a college degree or higher (Table 1). Consistent with previous studies [18, 34], the mean HEI was higher among individuals who consumed mushrooms compared to non-mushroom consumers (Table 1). During a mean 6.7 ± 3.4 y of follow-up (202,403 person-years), we identified a total of 2855 mortality cases. In the age- and sex-adjusted model (model 1), individuals with mushroom consumption had a lower risk of all-cause mortality compared with those without mushroom consumption (adjusted hazard ratio (HR) = 0.70; 95% CI: 0.56-0.88; Table 2). Additional adjustment for other potential confounding factors (model 3), including ethnicity-race, education status, marital status, BMI, smoking status, physical activity MET-min/week, alcohol, energy-adjusted carbohydrates, and fiber, total energy intakes, HEI-2010 score, the association between mushroom consumption and all-cause mortality was attenuated (adjusted HR = 0.84; 95% CI: 0.67-1.06; Table 2). When mushroom intake was further categorized into 4 groups, we did not observe a linear dose-response relationship between greater mushroom consumption and lower risk of all-cause mortality (*P-*trend = 0.23) (supplementary Figure 2). For cause-specific mortality, we did not find any statistically significant association between mushroom consumption and cardiovascular, cancer, Alzheimer’s, diabetes mellitus, and all other causes of mortality (data not shown).

**Table 1:** Weighted baseline characteristics of the study population, National Health and Nutrition Examination Survey (NHANES) 2003–2014 (*N* = 30,378)^a^

**Characteristic**	No Mushroom Intake (*n* = 29,033)	Mushroom Intake (*n* = 1345)	*P* value‡
Age, mean ± SE, years	45.8 ± 0.3	47.1 ± 0.7	0.07
Gender %			0.0004
Men	13,943 (47.8)	551 (41.4)	
Women	15,090 (52.2)	794 (58.6)	
Race-Ethnicity %			< 0.0001
Mexican American	5036 (8.9)	160 (5.0)	
Other Hispanic	2332 (4.9)	56 (1.8)	
Non-Hispanic White	13,109 (67.9)	857 (81.3)	
Non-Hispanic Black	6389 (12.0)	148 (5.4)	
Other Race	2167 (6.4)	124 (6.5)	
Education status %			< 0.0001
Non college degree	15,063 (42.3)	472 (30.6)	
College degree or above	13,970 (57.7)	873 (69.4)	
Marital status %			0.002
Married	16,788 (61.6)	872 (68.1)	
Not married	12,245 (38.4)	473 (31.9)	
Body mass index (kg/m^2^) %			0.007
< 24.9	8983 (32.3)	462 (37.3)	
25.0-29.9	9621 (33.0)	434 (31.9)	
≥ 30.0	10,429 (34.7)	449 (30.8)	
Smoking status %			0.72
Never smoker	15,943 (54.4)	719 (53.6)	
Ever smoker	13,090 (45.6)	626 (46.4)	
Physical activity MET-min/week			0.37
Somewhat active	6849 (22.5)	306 (21.7)	
Active	4562 (15.4)	243 (17.5)	
Very active	17,622 (62.1)	796 (60.8)	
Alcohol intake, (g/d)	8.9 ± 0.2	9.5 ± 0.7	0.38
Carbohydrate intake (g)/1000 kcal/d)	122.7 ± 0.3	117.9 ± 0.8	< 0.0001
Fiber intake (g)/1000 kcal/d	8.1 ± 0.1	8.8 ± 0.1	< 0.0001
Energy intake, kcal/d	2054 ± 7.5	2109 ± 26.0	0.04
Healthy Eating Index-2010	52.1 ± 0.2	55.8 ± 0.5	< 0.0001

Data are means ± SE unless indicated otherwise

*SE* Standard Error

‡For categorical variables, *P*-value was calculated by the Rao-Scott χ2 test, which is a design adjusted version of the Pearson χ2 test. For continuous variables, a t-test was used to calculate *P*-value

^a^All Ns are unweighted, and all proportions and means (SE) are survey-weighted for complex survey design to be nationally representative estimates. The focus should be on the survey-weighted proportions and means (SE) because they are representative of the US adult population

**Table 2:** Adjusted hazard ratios (95% confidence intervals) for all-cause mortality associated with mushroom intake (Yes/No), NHANES 2003–2014 (*N* = 30,378)

	No Mushroom Intake	Mushroom Intake
Person year (PY)	192,770	9633
Mortality case #	2740	115
Incidence rate (95% CI), per 1000 PY	14.2 (13.7, 14.8)	11.9 (9.9, 14.3)
Model 1	1(ref)	0.70 (0.56, 0.88)
Model 2	1(ref)	0.78 (0.63, 0.98)
Model 3	1(ref)	0.84 (0.67, 1.06)
**Sensitivity analyses**^**a**^		
Propensity score adjustment	1(ref)	0.85 (0.68, 1.07)
Excluding 594 deaths that occurred during the first 2 years of follow-up	1(ref)	0.90 (0.71, 1.15)
Excluding 12,955 participants with major chronic diseases (CVD, diabetes, and cancer)	1(ref)	0.72 (0.45, 1.16)

Model 1: Age and sex (men/women)-adjusted

Model 2: Model 1 + ethnicity-race (Mexican American, other Hispanic, non-Hispanic White, non-Hispanic Black, other race), education (non-college degree/college degree or above), marital status (never married/married) adjusted

Model 3: Model 2 + further adjustment of BMI (< 24.9, 25.0-29.9, ≥ 30), smoking status (never smoker, ever smoker), physical activity MET-min/week (somewhat active, active, very active), alcohol (g/d) intake, carbohydrates (g)/1000 kcal/d), fiber (g)/1000 kcal/d), total energy intake (kcal/d), healthy eating index-2010 score

^a^Based on model 3

In sensitivity analyses that adjusted for propensity scores or excluded mortality cases occurring in the first 2 years of follow-up or participants with a history of major chronic diseases, the results were similar to the original findings with no significant associations (Table 2). None of the interaction terms were found to be statistically significant (*P* for interaction > 0.05 for all).

### Meta-analysis results

We performed a meta-analysis by combining the continuous NHANES prospective study results with the findings of previous prospective cohort studies of mushroom intake and all-cause mortality. Our systematic literature search from January 1, 1966, up to May 1, 2021, identified 313 publications, including 170 articles from PubMed, 135 from Web of Science, and 8 from Cochrane Library databases, which two studies reported the association of mushroom consumption with mortality [34, 36]. In addition, we manually searched the bibliographies of one retrieved review [60] and found two more studies [35, 37]. Thus, including the present results from continuous NHANES, a total of 5 prospective cohort studies were included in the current meta-analysis. These studies included a total of 50,787 cases of deaths accrued in 601,893 men and women (Table 3). Two studies were conducted in the US, two in Japan, and one in Europe.

**Table 3:** Meta-analysis characteristics of included prospective cohort studies reporting mushroom consumption and risk of all cause-mortality

Author, year (reference)	Sex	Sample size, *n*	Dietary assessment	Outcome assessment	Country	Study design	Mean age (y)	Total Case, *n*	Mortality	Mushroom consumption (quantity)	Reported effect sizes: HR/RR (95% CI)	Follow-up, y)	Covariates in the fully-adjusted model
Ba et al. present study (2021)	M/F	Total:30,378 M:14,494; F:15,884	24-h recall	Death certificate	US	Cohort study (continuous NHANES)	45.9	2855	All-cause	Yes vs. No	HR: 0.84 (0.67, 1.06)	13	Age, sex, ethnicity-race, education, marital status, BMI, smoking status, physical activity, alcohol, carbohydrates, fiber, total energy intake, Healthy Eating Index-2010 score.
Ba et al. (2021) [34]	M/F	Total: 15,546 M:7047; F:8499	24-h recall	Death certificate	US	Cohort study (NHANES III)	44.3	5826	All-cause	Yes vs. No	HR: 0.84 (0.73, 0.98)	27	Age, sex, ethnicity-race, US regions, place of residence, education attainment, marital status, BMI, physical activity, total energy intake, fat, carbohydrates, fiber, alcohol, smoke 100+ cigarettes in life, Healthy Eating Index-2005 score.
Otsuka et al. (2020) [36]	M/F	Total: 799 M: 386; F: 413	3-day dietary record	National vital statistics records	Japan	Cohort study	68.1	289	All-cause	T3 (27.6 g/d) vs T1 (0 g/d)	HR: 0.97 (0.72, 1.32)	15.7	Age, sex, body mass index, education, smoking status, alcohol intake, physical activity, and history of hypertension, dyslipidemia, and diabetes mellitus
Leenders M et al. (2013) [35]	M/F	Total:451,151; M:129,882; F: 321,269	FFQ, dietary history, food record	Record linkage with cancer registries, boards of health, and death indices, health insurance records, cancer and pathology registries, next of kin	European countries: Denmark, France, Germany, Greece, Italy, the Netherlands, Norway, Spain, Sweden, and UK	Cohort study	51.2	25,682	All-cause	T3 vs. T1	HR: 0.94 (0.90, 0.98)	13	Age, smoking status, smoking duration, time since stopped smoking, number of cigarettes per day, alcohol, BMI, physical activity, education, processed meat, mutual adjustment between fruit and vegetables
Iso H et al. (2007) [37]	M/F	T: 104,019; M:43,850; F:60,169	Validated FFQ	Death rate among participants according to the responses to the diet questionnaire	Japan	Cohort study	Age 40-79 y	16,135	All-cause	Men ≥3 vs < 1/wk. Women ≥3 vs < 1/wk	RR: 0.97 (0.91, 1.03) RR: 0.96 (0.89, 1.03)	12.7	Age, area of study

**Fig. 1:**
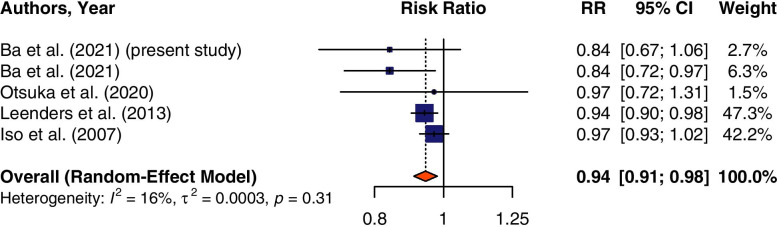
Summary forest plot of mushroom consumption and all-cause mortality risk. The square represents the point estimate of each study, and the size is proportional to its weight in the meta-analysis. The horizontal line through the square represents its 95% CI. The diamond represents the pooled risk ratio (RR) of the meta-analysis

There was an overall association between mushroom consumption and all-cause mortality using a random-effect model (pooled risk ratio: 0.94; 95% CI: 0.91, 0.98) (Fig. 1). There was no significant heterogeneity between studies (*I*^2^ = 15%; *P-*heterogeneity *p* = 0.32). Thus, no subgroup analysis was conducted.

## Discussion

Our meta-analysis of prospective cohort studies found that mushroom consumption was associated with a lower risk of all-cause mortality. In general, mushroom consumption is low in the US population [34, 61]. These findings emphasize the potentially significant clinical and public health implications of mushroom consumption in preventing premature mortality. In addition, the findings could be helpful in raising public health awareness about the potential health benefits of mushrooms and highlighting it as part of the healthy dietary patterns suggested in the Dietary Guidelines for Americans. The lack of observed significant association between mushroom consumption and all-cause mortality among NHANES participants could be attributed to the relatively smaller number of mortality cases among mushroom consumers in the continuous NHANES database, the type of consumed mushrooms in the US, or the use of 24-h dietary recalls data which could have underestimated the true association with the outcome. In addition, a previous study using NHANES III data found that mushroom consumption was associated with a lower risk of all-cause mortality [34]. Thus, the lack of significant association using continuous NHANES data could also be due to the difference in the covariate’s adjustments, such as regions and place of residence, which were not available in the continuous NHANES data. Furthermore, the length of follow-up was shorter for continuous NHANES compared with NHANES III (13 years vs. 27 years, respectively).

A recent systematic review and meta-analysis of observational studies conducted by our research team indicated that higher mushroom consumption was associated with a lower risk of total cancer, which could improve survivorship [34]. However, this inverse association was not consistently observed in other epidemiological studies that examined mushroom intake in relation to major chronic diseases, including cancer [62–65].

The protective effect of mushrooms against premature mortality may be related to their naturally high content of the potent antioxidants ergothioneine and glutathione. Oxidative stress occurs when there is an imbalance between oxidants (free radicals) and antioxidants defense systems, causing increases in oxidative damage, which has been linked to the pathogenesis of many chronic diseases and aging [66, 67]. Diets rich in antioxidants play a significant role in controlling and preventing chronic diseases [67]. Mushrooms are a rich source of potent antioxidants that can mitigate oxidative stress and improve human health [20]. Specifically, ergothioneine, an amino acid with a unique chemical structure, is found in very high levels in mushrooms and is obtained exclusively through dietary sources [7, 20, 27]. A recent study has suggested that higher plasma ergothioneine was associated with a lower mortality risk [68]. In addition, mounting pieces of evidence have indicated that ergothioneine is a “longevity vitamin” because of its multiple functions in the body (e.g., antioxidant, cytoprotective, and anti-aging) [69, 70]. Despite the benefits of ergothioneine, its consumption remains relatively low in the US, where its estimated dietary intake was the lowest compared to several other countries such as Italy and France [71]. Mushrooms are also a rich source of glutathione, which is considered to be a significant biological antioxidant, and its deficiency contributes to oxidative stress [20]. Additionally, mushrooms also contain other bioactive compounds, including chitin and polysaccharide β-glucans [5].

The potential benefits of establishing mushrooms as their own food group called “Fungi kingdom” or raising public awareness of mushrooms in other ways have been discussed [5, 72]. However, these efforts might have been limited by inadequate evidence from large-scale epidemiological studies that directly link dietary mushroom intake to major health outcomes.

Our study has several major strengths. We used a nationally representative sample of the US adult population. We also conducted the most comprehensive meta-analysis of prospective cohort studies for examining the association between mushroom consumption and the risk of all-cause mortality.

Notwithstanding, our study has some limitations that need to be addressed. First, mushroom consumption was estimated at baseline using two 24-h dietary recalls, which may not have adequately captured the actual usual intake. Such measurement error, if nondifferential, may have underestimated the association between mushroom intake and the risk of mortality. Second, information about the specific types of mushrooms consumed was not available in the NHANES database. Third, even though we controlled for major potential confounders including, demographics, major lifestyle, and dietary risk factors, including total energy in our final models, residual confounding is possible in observational studies. In addition, the adjustment factors used in the final models from each study were not the same for our meta-analysis, and non-NHANES studies did not adjust for the HEI. Lastly, we could not examine the dose-response relationship between mushroom consumption and mortality in the meta-analysis because not every study reported different levels of mushroom consumption per g/day.

Despite these limitations, this study provides insightful information about the potential role of mushroom intake in reducing the risk of premature mortality.

## Conclusions

The present findings of our meta-analysis provide more evidence about the benefits of mushroom consumption in reducing the risk of all-cause mortality. These findings can be used to support public health recommendations and increase awareness about the health-promoting effects of mushrooms. Large prospective cohort studies with repeated dietary data measurements are needed to replicate these findings and clarify the potential protective role of mushrooms against premature mortality.

## Supplementary Information

**Additional file 1: Supplemental Table 1.** Foods with Mushrooms identified by the USDA food code from the 24-h dietary recall, NHANES 2003-2014.

**Additional file 2: Supplemental Figure 1.** Flow Diagram.

**Additional file 3: Supplementary Figure 2.** Fully adjusted model 3 hazard ratios (HRs) of all-cause mortality risk associated with mushroom intake.

## Data Availability

The datasets used and/or analyzed during the current study are available at https://wwwn.cdc.gov/nchs/nhanes/.

## Abbreviations

NHANES: National Health and Nutrition Examination Survey
NCHS: National Center for Health Statistics
CDC: Centers for Disease Control and Prevention
FNDDS: Food and Nutrient Databases for Dietary Studies
DDC: Dietary Data Collection
ICD-10: The Tenth Revision of the International Classification of Diseases
HRs: Hazard ratios
CIs: 95% confidence intervals
SEs: Standard errors
US: United States
USDA: U.S. Department of agriculture
HEI: Healthy Eating Index
g: Grams
METs: Metabolic equivalent task
NDI: National Death Index
