# Supplementary material for: Prospective study of dietary mushroom intake and risk of mortality: results from continuous National Health and Nutrition Examination Survey (NHANES) 2003-2014 and a meta-analysis
Source: Nutr J. 2021 Sep 21;20:80. doi: 10.1186/s12937-021-00738-w (PMC8454070; doi:10.1186/s12937-021-00738-w)
Supplement: Supplementary file 1 — Additional file 1: Supplemental Table 1. Foods with Mushrooms identified by the USDA food code from the 24-h dietary recall, NHANES 2003-2014. [file 12937_2021_738_MOESM1_ESM.docx]

**Supplemental Table 1**. Foods with Mushrooms identified by the USDA food code from the 24-hour dietary recall, NHANES 2003-2014.

| **USDA Food Code** | **USDA Food Code Descriptions** |
| --- | --- |
| 27114000 | Beef with (mushroom) soup (mixture) |
| 27120090 | Ham or pork with (mushroom) soup (mixture) |
| 27144000 | Chicken or turkey with (mushroom) soup (mixture) |
| 27212400 | Beef and noodles with (mushroom) soup (mixture) |
| 27213400 | Beef and rice with (mushroom) soup (mixture) |
| 27213420 | Porcupine balls with (mushroom) soup (mixture) |
| 27220150 | Sausage and rice with (mushroom) soup (mixture) |
| 27242250 | Chicken or turkey and noodles with (mushroom) soup (mixture) |
| 27243400 | Chicken or turkey and rice with (mushroom) soup (mixture) |
| 27250124 | Shrimp and noodles with (mushroom) soup (mixture) |
| 27250630 | Tuna noodle casserole with (mushroom) soup |
| 27250710 | Tuna and rice with (mushroom) soup (mixture) |
| 27250830 | Fish and rice with (mushroom) soup |
| 27250900 | Fish and noodles with (mushroom) soup |
| 27311610 | Beef, potatoes, and vegetables (including carrots, broccoli, and/or dark-green leafy), (mushroom) soup (mixture) |
| 27311620 | Beef, potatoes, and vegetables (excluding carrots, broccoli, and dark-green leafy), (mushroom) soup (mixture) |
| 27350410 | Tuna noodle casserole with vegetables and (mushroom) soup |
| 27450510 | Tuna casserole with vegetables and mushroom sauce, no noodles |
| 27510260 | Cheeseburger, 1/4 lb meat, with mushrooms in sauce, on bun |
| 28316020 | Beef and mushroom soup, canned, low sodium |
| 28345160 | Chicken and mushroom soup, cream of, prepared with milk |
| 28500100 | Gravy, mushroom |
| 32105048 | Egg omelet or scrambled egg, with mushrooms |
| 32105070 | Egg omelet or scrambled egg, with mushrooms |
| 32105130 | Egg omelet or scrambled egg, with onions, peppers, tomatoes, and mushrooms |
| 58304050 | Spaghetti with meat and mushroom sauce (diet frozen meal) |
| 72201240 | Broccoli, cooked, NS as to form, with mushroom sauce |
| 72201241 | Broccoli, cooked, from fresh, with mushroom sauce |
| 72201242 | Broccoli, cooked, from frozen, with mushroom sauce |
| 72202020 | Broccoli casserole (broccoli, rice, cheese, and mushroom sauce) |
| 75115000 | Mushrooms, raw |
| 75219000 | Mushrooms, cooked, NS as to form, NS as to fat added in cooking |
| 75219001 | Mushrooms, cooked, from fresh, NS as to fat added in cooking |
| 75219002 | Mushrooms, cooked, from frozen, NS as to fat added in cooking |
| 75219010 | Mushrooms, cooked, NS as to form, fat not added in cooking |
| 75219011 | Mushrooms, cooked, from fresh, fat not added in cooking |
| 75219012 | Mushrooms, cooked, from frozen, fat not added in cooking |
| 75219013 | Mushrooms, cooked, from canned, fat not added in cooking |
| 75219020 | Mushrooms, cooked, NS as to form, fat added in cooking |
| 75219021 | Mushrooms, cooked, from fresh, fat added in cooking |
| 75219022 | Mushrooms, cooked, from frozen, fat added in cooking |
| 75219023 | Mushrooms, cooked, from canned, fat added in cooking |
| 75219100 | Mushroom, Oriental, cooked, from dried |
| 75315210 | Peas with mushrooms, cooked, fat not added in cooking |
| 75340130 | Vegetable combination (green beans, broccoli, onions, mushrooms), cooked, NS as to fat added in cooking |
| 75340140 | Vegetable combination (green beans, broccoli, onions, mushrooms), cooked, fat not added in cooking |
| 75340150 | Vegetable combination (green beans, broccoli, onions, mushrooms), cooked, fat added in cooking |
| 75402020 | Beans, lima, immature, cooked, NS as to form, with mushroom sauce |
| 75403020 | Beans, string, green, cooked, NS as to form, with mushroom sauce |
| 75403022 | Beans, string, green, cooked, from frozen, with mushroom sauce |
| 75403023 | Beans, string, green, cooked, from canned, with mushroom sauce |
| 75414010 | Mushrooms, NS as to form, creamed |
| 75414011 | Mushrooms, from fresh, creamed |
| 75414013 | Mushrooms, from canned, creamed |
| 75414020 | Mushrooms, stuffed |
| 75414030 | Mushrooms, batter-dipped, fried |
| 75417021 | Peas, cooked, from fresh, with mushroom sauce |
| 75505000 | Mushrooms, pickled |
| 75607000 | Mushroom soup, NFS |
| 75607010 | Mushroom soup, cream of, prepared with milk |
| 75607020 | Mushroom soup, cream of, prepared with water |
| 75607030 | Mushroom soup, canned, undiluted |
| 75607040 | Mushroom soup, with meat broth, prepared with water |
| 75607050 | Mushroom soup, cream of, low sodium, prepared with water |
| 75607060 | Mushroom soup, cream of, NS as to made with milk or water |
| 75607080 | Mushroom with chicken soup, cream of, prepared with milk |
| 75607090 | Mushroom soup, cream of, canned, reduced sodium, NS as to made with milk or water |
| 75607100 | Mushroom soup, cream of, canned, reduced sodium, prepared with milk |
