# Supplementary material for: Prospective study of dietary mushroom intake and risk of mortality: results from continuous National Health and Nutrition Examination Survey (NHANES) 2003-2014 and a meta-analysis
Source: Nutr J. 2021 Sep 21;20:80. doi: 10.1186/s12937-021-00738-w (PMC8454070; doi:10.1186/s12937-021-00738-w)
Supplement: Supplementary file 2 — Additional file 2: Supplemental Figure 1. Flow Diagram. [file 12937_2021_738_MOESM2_ESM.docx]

**Identification**

Records identified through databases searching and manual search of reference lists:

Pubmed, Web of Science, and Cochrane Library

(*n*=313)

Excluded duplicate studies (*n*=89)

**Screening**

Records excluded after title/abstracts screening (*n*=139)

Records screened (*n*=224)

**Eligibility**

Full-text articles excluded, with reasons (*n*=81)

-Meta-analysis/reviews (*n*=25)

-Not relevant exposures and/or outcomes not reported, or not

prospective design (*n*=56)

Articles included in qualitative synthesis (*n*=4)

Full-text articles assessed

for eligibility

(*n*=85)

**Included**

Articles included in quantitative synthesis plus present study (meta-analysis)

(*n*=5)
