# Supplementary material for: Prospective study of dietary mushroom intake and risk of mortality: results from continuous National Health and Nutrition Examination Survey (NHANES) 2003-2014 and a meta-analysis
Source: Nutr J. 2021 Sep 21;20:80. doi: 10.1186/s12937-021-00738-w (PMC8454070; doi:10.1186/s12937-021-00738-w)
Supplement: Supplementary file 3 — Additional file 3: Supplementary Figure 2. Fully adjusted model 3 hazard ratios (HRs) of all-cause mortality risk associated with mushroom intake. [file 12937_2021_738_MOESM3_ESM.pdf]

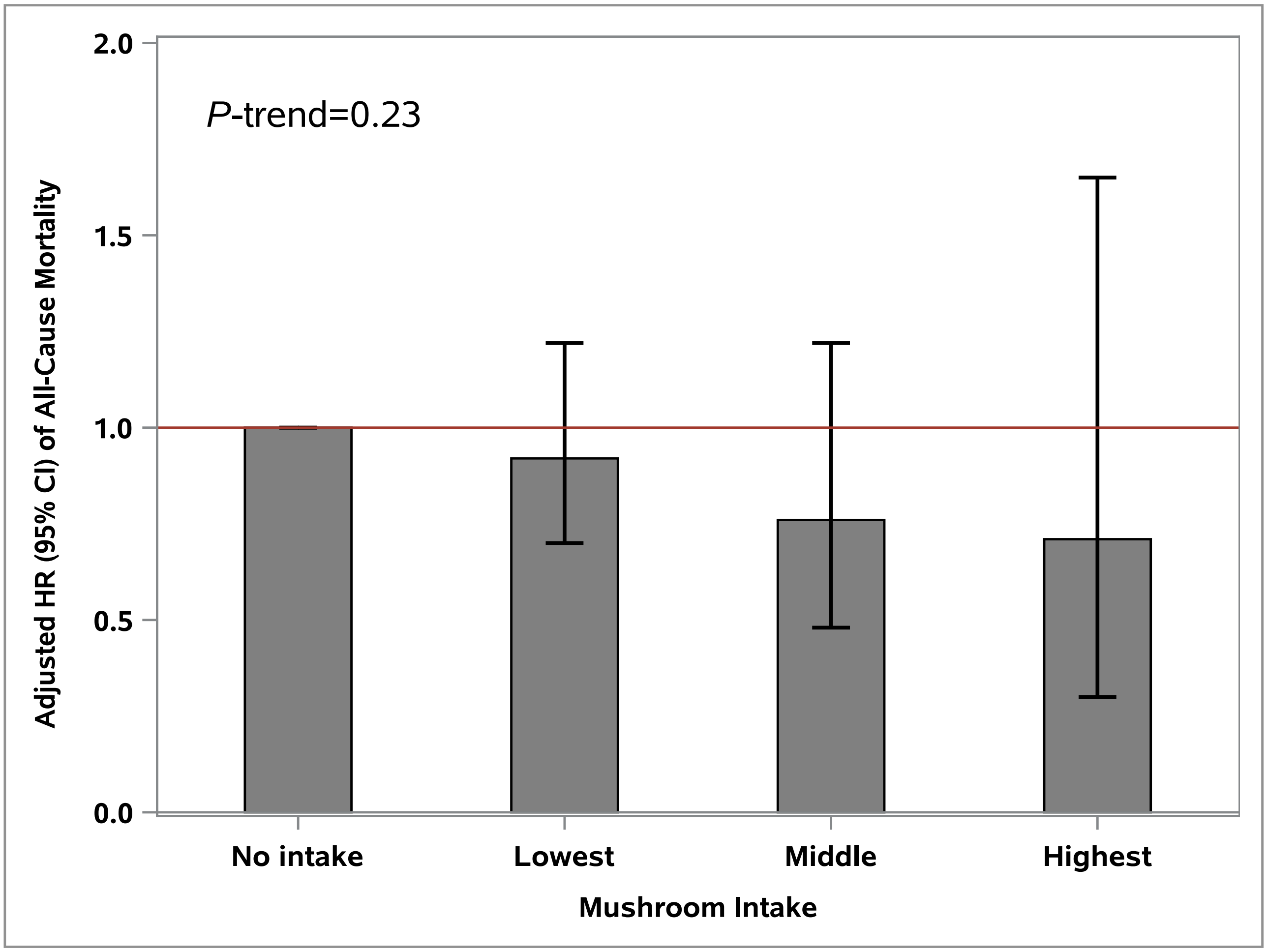

Supplementary Figure 2. Fully adjusted model 3 hazard ratios (HRs) of all-cause mortality risk associated with mushroom intake. Error bars represent 95% confidence intervals.
